# SARS-CoV-2 updates in a West African population and precautionary measures for sustaining quality antenatal care delivery

**DOI:** 10.7189/jogh.10.020365

**Published:** 2020-12

**Authors:** Emmanuel Komla Senanu Morhe, Enoch Odame Anto, David Antony Coall, Eric Adua, Alexander Yaw Debrah, Otchere Addai-Mensah, Michael Owusu, William KBA Owiredu, Christian Obirikorang, Emmanuel Akomanin Asiamah, Emmanuel Acheampong, Evan Adu Asamoah, Lydia Abradu, Agartha Odame Anto, Youxin Wang, Haifeng Hou, Wei Wang

**Affiliations:** 1 School of Medicine, Department of Obstetrics and Gynecology, University of Health and Allied Sciences, Volta Region, Ghana, West Africa; 2 Department of Obstetrics and Gynecology, Ho Teaching Hospital, Volta Region, Ghana, West Africa; 3 College of Health Sciences, Department of Medical Diagnostic, Kwame Nkrumah University of Science and Technology, Kumasi, Ghana, West Africa; 4 School of Medical and Health Sciences, Edith Cowan University, Perth, Australia; 5 Kumasi Centre for Collaborative Research in Tropical Medicine, Kumasi, Ghana, West Africa; 6 School of Medicine and Dentistry, Department of Molecular Medicine, Kwame Nkrumah University of Science and Technology, Kumasi, Ghana, West Africa; 7 School of Health and Allied Sciences, Department of Medical Laboratory Sciences, University of Health and Allied Sciences, Volta Region, Ghana, West Africa; 8 School of Nursing and Midwifery, Department of Obstetrics and Gynecology, University of Health and Allied Sciences, Volta Region, Ghana, West Africa; 9 Beijing Key Laboratory of Clinical Epidemiology, School of Public Health, Capital Medical University, Beijing, China; 10 School of Medical and Health Sciences, Shandong First Medical University, Shandong, China

The coronavirus disease 2019 (COVID-19) is currently a global health burden characterised by Severe Acute Respiratory Syndrome Coronavirus 2 (SARS-CoV-2) infections [1]. Since it was declared as a Public Health Emergency by the World Health Organisation (WHO), the virus has infected a total of 57 870 Africans with 2154 deaths and 19 363 recoveries as at 9 May 2020 (**Figure 1**, panel A) [4]. Of these infections, Ghana has recorded 4012 cases, positioning her as the 5th most infected country in Africa and 1st most infected country in West Africa [2,3].

**Figure 1:**
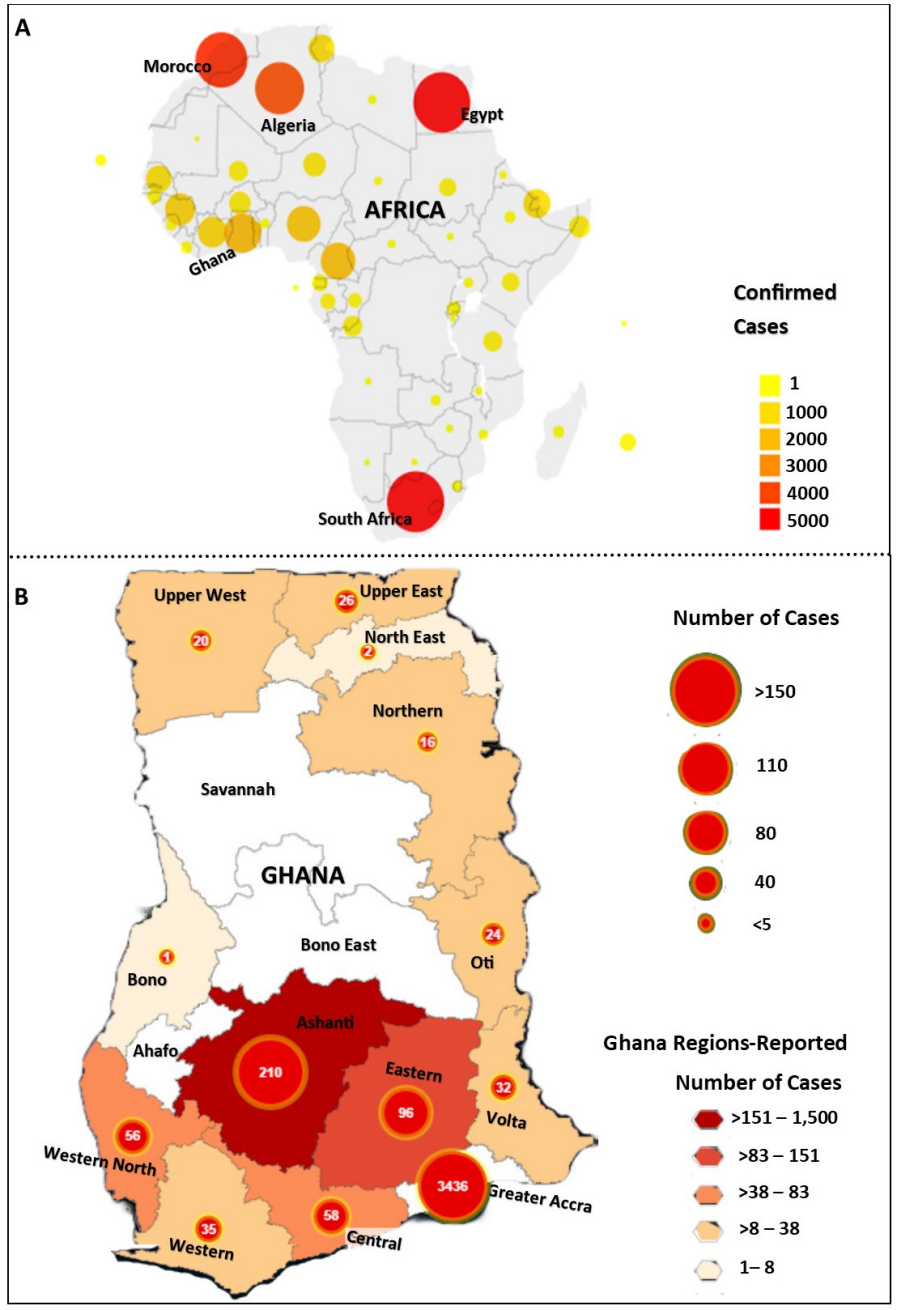
Update of COVID-19 cases in Africa and the regional distribution of cases in Ghana. **Panel A.** Distribution of confirmed COVID-19 cases in Africa with Ghana recording the 5th highest number of cases. **Panel B.** Regional distribution of cases in Ghana (**B**) as at 9 May 2020. Maps were downloaded from the African Argument Organisation reports [2] and Ghana Health Services updates [3] linked to the COVID-19 data repository of the Johns Hopkins Center for Systems Science and Engineering on 9 May 2020.

Ghana’s first two cases of COVID-19 infections were confirmed on 12 March 2020 [3,5]. These cases occurred following the entry or return of travellers from heavily infected countries in Europe, America and neighbouring West African countries in March [3,5]. As of 9 May 2020, the positivity rate of COVID-19 infection in Ghana is 2.7% (4012 out of 149 948 tested cases) with 18 (0.4%) deaths, 323 (8.1%) recoveries and 6 (0.1%) in critical conditions undergoing intensive care [3,5]. All total deaths, however, were reported to have had serious underlying chronic conditions. Of the16 administrative regions in Ghana, 13 have reported cases with the Greater Accra Region reporting the majority of cases (85.6%) as at 9 May 2020 (**Figure 1**, panel B) [3,5]. The slow increase in Ghana’s cases is due to the appropriate preventive measures adopted by the Ghana Health Service (GHS) and the Ministry of Health (MOH) in line with recommendations from the WHO and closure of all borders enforced by the Ghana Immigration Services to effectively undertake community enhanced surveillance, mandatory quarantining all international travellers and mandatory self-quarantine for those who had come into close contact with affected individuals. This was done to achieve the 3Ts ie, enhanced contact tracing, testing to identify cases of infected persons and then isolating them for treatment to curtail the spread of the virus [5]. Ghana is among the leading countries in Africa reported to have tested the highest numbers of suspected cases via Real-Time Polymerase Chain Reaction technique from the two major research centres, ie, the Noguchi Memorial Institute for Medical Research and the Kumasi Centre for Collaborative Research. In Ghana, males (61%) have recorded the highest rates of infection, whereas pregnant women and children (<15 years), albeit minimal, has also been reported [3,5].

## THE SITUATION OF PREGNANT MOTHERS AND BABIES IN GHANA

In Ghana, two cases of COVID-19 have publicly been confirmed for pregnant women out of the 4012 total positive cases recorded as at 9 May 2020.

The first case was a 22-year pregnant woman who travelled from the Greater Accra region to the Volta region to attend the antenatal care clinic and later delivered through caesarean section at the Hohoe Municipal Hospital in the Volta region but was subsequently found to be positive for COVID-19 after delivery [3]. After close monitoring and treatment, she has recovered, and the baby is feeding well with no mother-to-baby transmission of the virus. This is consistent with previous reports in Wuhan, China [6,7]. The 22-year-old pregnant woman was one of the first two positive cases recorded in the Volta region after mandatory quarantine and testing of about 40 hospital staff members in the Hohoe Municipal Hospital and 85 community persons who came into close contact with her. Even though there are 172 Health facilities in the Volta Region, the Ho Teaching Hospital (HTH) Isolation Centre and the Ho Medical Village and Innovation Centre at Kpetoe are the only designated isolation and treatment centres for all suspected and confirmed COVID-19 cases in the Volta Region of Ghana [5].

The second case was a 34-year-old mother in her ninth month of pregnancy who attended an antenatal care clinic at the Bolgatanga Regional Hospital in the Upper East region with complaints of sore throat and showing symptoms of COVID-19 after being admitted to the maternity ward. She later tested positive for COVID-19, becoming the first cases in the Upper East Region. She has currently recovered after isolation and treatment at the Isolation centre at the Bolgatanga Regional Hospital.

Nevertheless, there have been several concerns from the Ghanaian community suggesting that pregnant women may be susceptible to the COVID-19 since some symptoms of early morning sickness such as gastrointestinal symptoms, fatigue and body weakness are somewhat comparable to SARS-CoV-2. Early morning sickness, however, does not present with fever as in the case of COVID-19 but is a common symptom of early pregnancy. Even though pregnant women are currently not recognised as the high-risk population for COVID-19 or SARS-CoV-2, the demand of pregnancy may cause immunological and physiological changes that may make pregnant mothers more vulnerable to viral respiratory infections, such as influenza associated with severe acute respiratory syndrome (SARS) [8,9]. A recent report in Beijing, China, however, indicated that the prevalence of SARS-CoV-2 in pregnancy is extremely low and case fatalities are few even in the presence of underlying morbidities [10].

Amid the COVID-19 pandemic, one of the major concerns of the Ghana Health Service (GHS) and the Ministry of Health (MOH) is to maintain quality antenatal care (ANC). Attending regular ANC clinics is important for identifying and reducing the risk of adverse complications to the mother and the growing foetus. In developing countries such as Ghana, there is, therefore, a need to maintain quality ANC services and increase resource availability where possible amid this pandemic.

## WHAT IS BEING DONE FOR PREGNANT MOTHERS AND BABIES IN GHANA?

**Figure:**
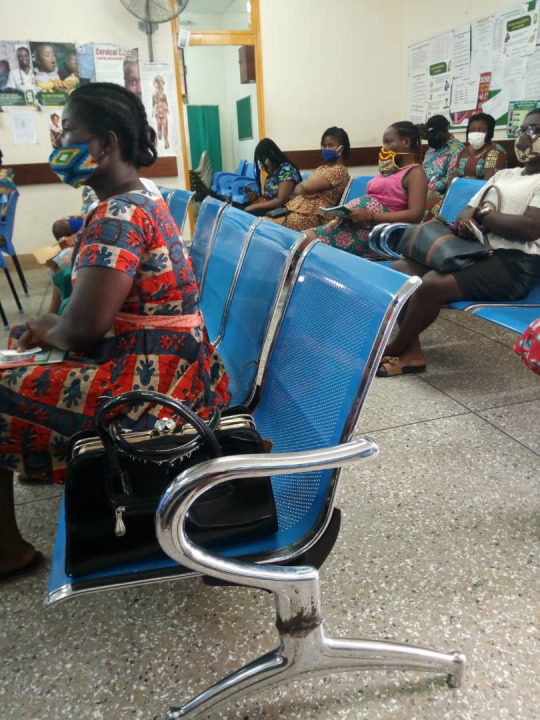
Photo: A group of Ghanaian pregnant women at the Ho Teaching Hospital (HTH) wearing masks and adhering to social distancing (Photographer: Agartha Odame Anto from HTH).

In this viewpoint, we briefly describe the precautions taken by the Ho Teaching Hospital (HTH) management as instituted by the Ministry of Health and the Ghana Health Service in line with recommendations from the WHO to limit the spread of the virus. Specifically, we looked at the precautionary measures taken to maintain quality antenatal care (ANC) delivery in the HTH, Ghana.

For example, the management of HTH has advised parents of clients not to visit the hospital unless it is absolutely necessary. Activities that allowed mass gatherings such as face-to-face teaching and in-service training programs by medical and nursing students have been suspended. Regarding the ANC unit, the HTH management created a larger open space area and relocated our daily antenatal clinic initial procedures to this area ensuring all social distancing requirements were maintained. Also, all forms of visits by patients´ relatives have been banned and restricted entry to only a limited number of parents who may need to assist pregnant women with challenging conditions or provide legal authoritative notes on behalf of the pregnant mothers.

Training on Infection Prevention and Control (IPC) measures have been provided to all the hospital staff. Handwashing and sanitisation opportunities in and around the main entrance of the hospital and its units have been provided for staff, patients and parents. The mandatory check for high temperature before entry into both HTH and specialised units such as the antenatal clinic was enforced. As of Monday, 27 April 2020, the wearing of masks in Ghana became mandatory and we have adopted this protocol at the HTH. The government of Ghana continues to support the mass production of masks by the local industries to ensure that sufficient quantities are available to all hospital staff and frontline health workers for free, and to the general public at an affordable price.

We, however, anticipate a potential drawback of these preventive measures due to the economic hardship to be faced by the Ghana governments, the increase in the cost of preventive maintenance, labour and over-maintenance during the COVID-19 period. Consequently, the restriction of entry for parents/family impedes the drive to promote family-centred care in the antenatal care unit and will impede bonding between the pregnant women, their newborns and husbands as well as prevent other practical, social, emotional and spiritual support they receive from other relatives. Also, we foresee a shortage of supplies for essential antenatal care as the Nation and HTH divert resources towards the COVID-19 response.

We are, however, optimistic that the provision of precautionary measures will be significant in the pursuit of disease prevention and to provide quality ANC during and beyond the pandemic. Although challenging and with unexpected benefits, the COVID-19 pandemic has not only reawakened observance to IPC protocols in antenatal care but also provided insights on actions to adhere to social distancing in an overcrowded unit. We are exploring the feasibility of home-based mobile-health and telemedicine technologies in addition to improved client education to ensure continuity of care for our vulnerable pregnant women. We, however, expect that low resource availability may impede the progress of health care, especially in rural communities. Thus, the Government of Ghana is currently preparing to provide additional 88 district hospital facilities, and 8 isolation and laboratory testing centres to overcome this challenge [5].

## CONCLUSIONS

We found two confirmed case of COVID-19 among pregnant women in Ghana who have recovered with no mother-to-baby transmission as of 9 May 2020. Nevertheless, restrictions imposed to contain the spread are already impacting positively on antenatal care delivery in Ghana. With continued close attention, it is not likely that maternal mortality related to COVID-19 will occur, however, the risk may increase, if the pandemic prolongs. Therefore, there is a need to continue improving upon our health care setting by maintaining precautionary measures to contribute to the quality of ANC delivery.
